# Machine learning models to predict the warfarin discharge dosage using clinical information of inpatients from South Korea

**DOI:** 10.1038/s41598-023-49831-6

**Published:** 2023-12-18

**Authors:** Heejung Choi, Hee Jun Kang, Imjin Ahn, Hansle Gwon, Yunha Kim, Hyeram Seo, Ha Na Cho, JiYe Han, Minkyoung Kim, Gaeun Kee, Seohyun Park, Osung Kwon, Jae-Hyung Roh, Ah-Ram Kim, Ju Hyeon Kim, Tae Joon Jun, Young-Hak Kim

**Affiliations:** 1 Department of Medical Science, Asan Medical Institute of Convergence Science and Technology, Asan Medical Center, University of Ulsan College of Medicine, 88, Olympic-ro 43 gil, Songpa-gu, Seoul, 05505 Republic of Korea; 2 Division of Cardiology, Asan Medical Center, 88, Olympicro 43gil, Songpagu, Seoul, 05505 Republic of Korea; 3 Department of Information Medicine, Asan Medical Center, 88, Olympicro 43gil, Songpagu, Seoul, 05505 Republic of Korea; 4 Division of Cardiology Department of Internal Medicine, Eunpyeong St Mary’s Hospital, Catholic University of Korea, Seoul, Republic of Korea; 5 Department of Internal Medicine, Chungnam National University College of Medicine, Chungnam National University Sejong Hospital, 20, Bodeum 7-ro, Sejong-si, 30099 Sejong Republic of Korea; 6 Division of Cardiology, Department of Internal Medicine, Asan Medical Center, University of Ulsan College of Medicine, Seoul, Republic of Korea; 7 Department of Cardiology, Cardiovascular Center, Korea University Anam Hospital, Korea University College of Medicine, 73, Goryeodae-ro, Seongbuk-gu, Seoul, 02841 Republic of Korea; 8 Big Data Research Center, Asan Institute for Life Sciences, Asan Medical Center, 88, Olympicro 43gil, Songpagu, Seoul, 05505 Republic of Korea; 9 Division of Cardiology, Department of Information Medicine, Asan Medical Center, University of Ulsan College of Medicine, 88, Olympicro 43gil, Songpagu, Seoul, 05505 Republic of Korea

**Keywords:** Health care, Biomedical engineering, Computer science

## Abstract

As warfarin has a narrow therapeutic window and obvious response variability among individuals, it is difficult to rapidly determine personalized warfarin dosage. Adverse drug events(ADE) resulting from warfarin overdose can be critical, so that typically physicians adjust the warfarin dosage through the INR monitoring twice a week when starting warfarin. Our study aimed to develop machine learning (ML) models that predicts the discharge dosage of warfarin as the initial warfarin dosage using clinical data derived from electronic medical records within 2 days of hospitalization. During this retrospective study, adult patients who were prescribed warfarin at Asan Medical Center (AMC) between January 1, 2018, and October 31, 2020, were recruited as a model development cohort (n = 3168). Additionally, we created an external validation dataset (n = 891) from a Medical Information Mart for Intensive Care III (MIMIC-III). Variables for a model prediction were selected based on the clinical rationale that turned out to be associated with warfarin dosage, such as bleeding. The discharge dosage of warfarin was used the study outcome, because we assumed that patients achieved target INR at discharge. In this study, four ML models that predicted the warfarin discharge dosage were developed. We evaluated the model performance using the mean absolute error (MAE) and prediction accuracy. Finally, we compared the accuracy of the predictions of our models and the predictions of physicians for 40 data point to verify a clinical relevance of the models. The MAEs obtained using the internal validation set were as follows: XGBoost, 0.9; artificial neural network, 0.9; random forest, 1.0; linear regression, 1.0; and physicians, 1.3. As a result, our models had better prediction accuracy than the physicians, who have difficulty determining the warfarin discharge dosage using clinical information obtained within 2 days of hospitalization. We not only conducted the internal validation but also external validation. In conclusion, our ML model could help physicians predict the warfarin discharge dosage as the initial warfarin dosage from Korean population. However, conducting a successfully external validation in a further work is required for the application of the models.

## Introduction

Warfarin is an oral anticoagulant; it has been used for the treatment and prevention of thromboembolic disorders for more than 60 years^1^. Despite its well-studied clinical pharmacology, high efficacy, and cost-effectiveness, it is clinically challenging to determine the appropriate dosage of warfarin for each individual because of its narrow therapeutic window and variable patient responses^2^. Warfarin is one of the ten main anticoagulants that cause adverse drug events^3^. The risk of thrombosis increases if the dosage is insufficient, and conversely, the risk of bleeding increases if the dosage is excessive^3^. Conventionally, the international normalized ratio (INR) blood coagulation test is performed daily to achieve optimal efficacy and minimize side effects of warfarin, and physicians adjust the warfarin dosage individually based on their medical experience and INR values^4^.

Recent studies have attempted to predict the ideal warfarin dosage using machine learning algorithms, and most models were developed using genetic data obtained through genetic testing^5–12^. Genetic variations in CYP2C9, VKORC1, and CYP4F2 have been shown to have significant correlations with warfarin. However, genetic testing is not performed in actual clinical settings because it is time-consuming^13^. One study aimed to predict the adjustment dose of warfarin using only clinical data^14^. However, warfarin adjustment doses are prescribed to outpatients and can interact with some foods or alcohol, thus affecting their lifestyle. In such cases, it is questionable whether it is more appropriate to use lifelog data that reflects the patient’s lifestyle and environment than using clinical data included in the electronic medical records. Consequently, it is more proper to use EMR derived from controlled inpatients populations in large hospitals for initial warfarin dose prediction.

In this study, we suggested that clinical warfarin dosing algorithms that predict discharge warfarin dosage to the initial warfarin dosage without genetic data are important for the following rationale. In tertiary hospital, it is difficult to use the genetic test in initial warfarin dose judgement immediately after hospitalized, becase it takes about two weeks the genetic test to come out. It results in physicians must decide the initial warfarin dosage based on limited the patient’s information. Accordingly, we concluded that a tool providing reliable warfarin dosage from limited clinical information is clinically relevant.

Finally, we proposed machine learning models that predict the warfarin discharge dosage as an appropriate initial warfarin dosage using only clinical data generated in the hospital within 2 days of hospitalization. The models proposed in this study can not only reduce unnecessary treatment duration but also prevent the adverse drug event of warfarin, by rapidly presenting appropriate warfarin dosing. Additionally, it can contribute to both hospitals and patients, by securing space for other patients in hospital wards and reducing the financial burden of hospitalization costs for patients.

## Methods

### Ethical approval

The Institutional Review Board of Asan Medical Center approved the protocols of this study (No. 2021-0321), which was conducted corresponding to the 2008 Declaration of Helsinki. Also, this study exempted the requirement for informed consent as the database used for the study consisted of anonymous, de-identified information. All experiments were performed in accordance with relevant guidelines and regulations. Also, we gained the data access to MIMIC-III database to use for external validation process taking courses titled *Biomedical Responsible Conduct of Research* from CITI.

### Study design

**Figure 1:**
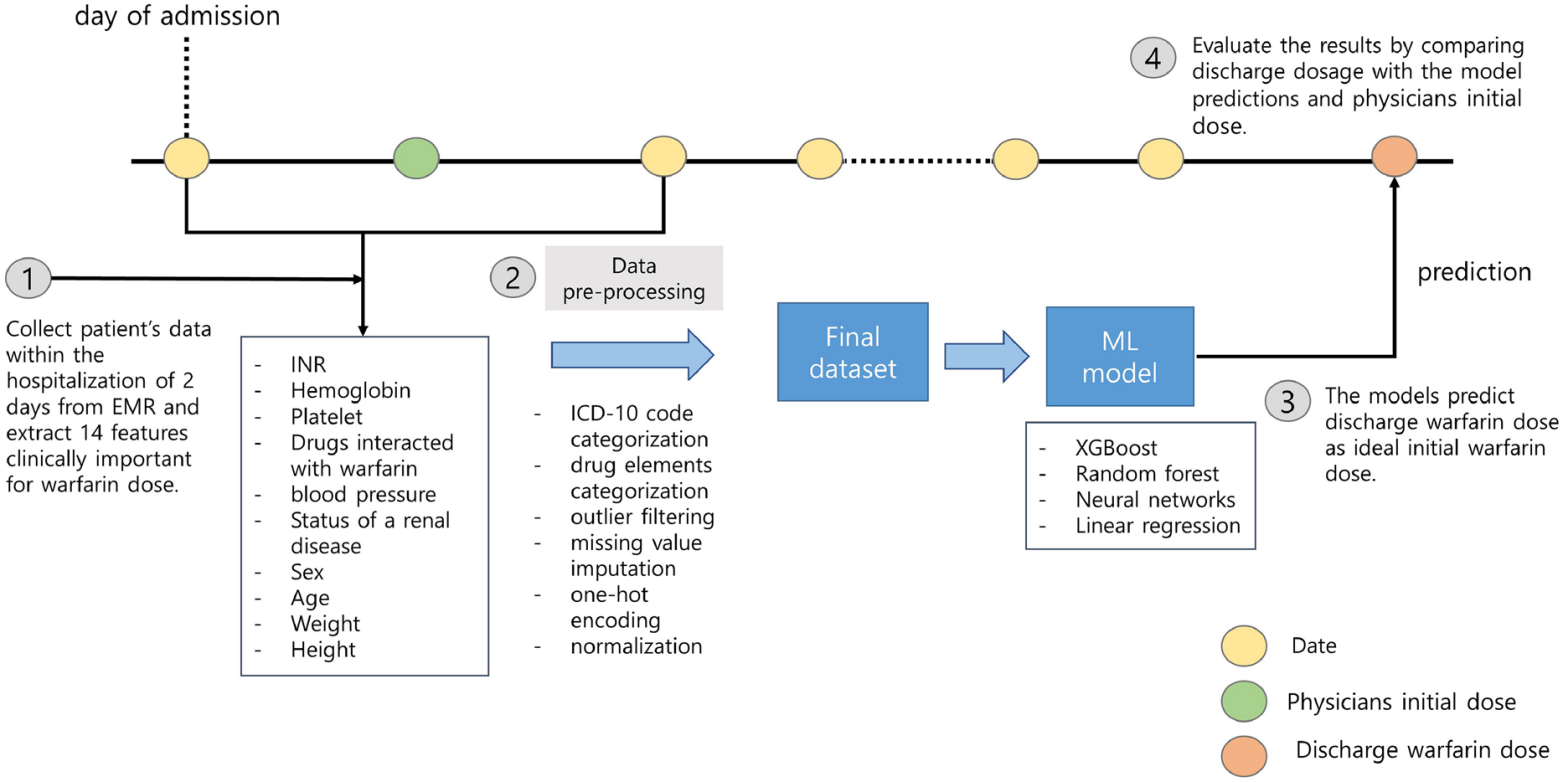
Overview of the workflow. Our single-center study complied with the workflow in the following order: (1) based on clinical rationales, we extracted 17 features associated with warfarin from the electronic medical record (EMR) database up to the second day of hospitalization; (2) we conducted data pre-processing, such as missing value imputation, outlier filtering, and normalization, and created the final dataset; (3) the models that predict the warfarin discharge dosage using the dataset were developed. (4) The discharge dose was used as ground-truth to calculate the error with physicians initial dose and model predictions. The yellow circles represent the one day during hospitalization. The green and orange circle indicate the first warfarin dosage after hospitalization and the warfarin dosage at discharge, respectively.

In general, warfarin is prescribed at 8 p.m based on initial blood test results, physiological measurements, and concomitant medications of a patient on the day of starting warfarin. Initially, a dose of 5 mg or higher may be initiated to rapidly achieve a therapeutic level, but in cases where the patient is deemed to be at a higher risk of bleeding, a lower dose of 1–2 mg may be used. Following the initiation of warfarin, the daily INR tests are performed to determine the appropriate dosage until the patient reaches a stable therapeutic level (INR 2.0–3.0). While some patients may easily reach the appropriate warfarin dosage, for others, the daily fluctuations in INR values can lead to several days of adjustment before achieving the target range of INR 2.0–3.0. Among the patients admitted to Asan Medical Center, those included in this dataset typically undergo such a process. Reflecting the warfarin therapy schedule of the patients, we designed four machine learning models to predict the warfarin dosage prescribed at discharge using only clinical data measured on the first and second days of hospitalization (Fig. 1). At first, we extracted clinical variables in electronic medical records (EMRs) of the Asan Medical Center (AMC) and Medical Information Mart for Intensive Care III (MIMIC-III) database^15^ with same criteria for model development and validation. Then, data pre-processing was performed and the models were trained using training set. To assess the clinical utility of the models, the initial warfarin dosage prescribed by physicians was compared with the models in terms of the mean absolute error (MAE)^16^ and accuracy. Finally, we evaluated our models in the internal validation set that whether the models can provide more accurate warfarin dosage than physicians initial dose. In addition, we externally validated the models to external validation set derived from the MIMIC-III. Data pre-processing, model development, training and validation were conducted in Python 3.8.10. Additionally, we analyzed the predictions of five physicians for 40 data points by calculating the intraclass correlation coefficient (ICC)^17^ value and compared with the distributions of the model predictions to explore the objectivity of the models.

### Data source

We used the EMRs of the Asan Medical Center (AMC) as the source of development cohort. The EMRs was derived from Asan BiomedicaL Research Enviroment (ABLE) platform that Asan Medical Center has been developing a de-identification system for biomedical research^18^. It ensured the accuracy and completeness of the data.

### Data collection

**Figure 2:**
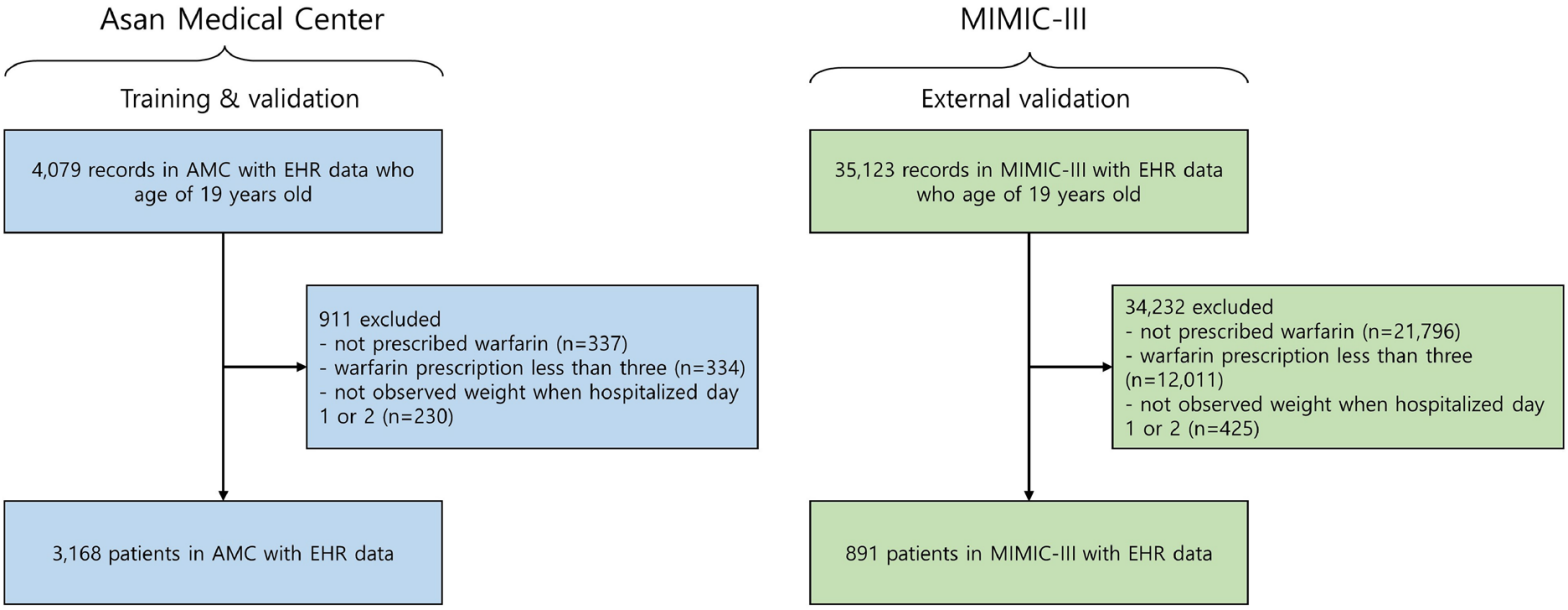
Cohort diagram. *AMC* Asan medical center, *EHR* electronic health record, *MIMIC-III* medical information mart for intensive care III.

The model development cohort consisted of patients admitted to the cardiovascular or thoracic and cardiovascular surgery departments of AMC between January 1, 2018, and October 31, 2020. All the selected participants were at least 19 years old; exclusions were based on the following criteria: none of warfarin prescription at discharge;< 3 warfarin prescriptions; and no weight measurements within 2 days of hospitalization (Fig. 2). The external validation set derived from the MIMIC-III followed the same workflow except for medical department codes, as the medical department codes of the intensive care unit (ICU) can not be found. Finally, the development cohort derived from AMC EMRs comprised 3168 patients and the external validation cohort derived from the MIMIC-III comprised 891 patients.

### Feature selection

The process of feature selection used for model development and validation was conducted only when had already been proofed correlations with warfarin based on clinical rationales^19–26^. The 17 clinical variables regarded as key factors of warfarin dosage adjustments and associated with bleeding were selected from the four tables of demographic, diagnosis, medication, vital sign (Supplementary Table S1). First of all, four demographic variables of age, sex, height, weight were used. Second, disease status of renal disease was selected in the diagnosis table, due to association with renal disease and warfarin. Third, seven medicines, affecting functions of warfarin and increasing a risk of bleeding and causing warfarin dosage adjustments when combined with warfarin, were selected in the medication table. Additionally, systolic blood pressure and diastolic blood pressure measurements were selected as variables in the vital sign table because uncontrolled blood pressures can increase bleeding.

### Categorization

We conducted categorization of variables in the status of renal disease and concurrent medication twice to reduce redundancy and consider more comprehensive information. It allowed us to group variables with clinical associations together and capture their collective impact on the prediction. This approach made the machine learning models more robust exploring broader patterns and relationships in the dataset. At first, the International Classification of Diseases, Tenth Revision (ICD-10) codes, which are the diagnostic codes used in the AMC EMRs, have a hierarchical structure; therefore, they can be grouped using the first disease category code. For example, code I48 includes all diseases related to atrial fibrillation and flutter. Finally, all ICD-10 codes for renal disease are selected based on the first three or four characters and can be found as Supplementary Table S2. Then, the medication information was categorized into each group based on the associated ingredients of the prescribed drugs. In specific, rosuvastatin, simvastatin, and atorvastatin were grouped as statins, whereas furosemide and torasemide were grouped as diuretic (loop). Last, isosorbide dinitrate and nitroglycerin were grouped as nitrate. Consequently, a total of 35 components were assigned into 7 groups with similar drug effects.

### Data transformation

The categorical variables need to convert into numerical values because it can’t use in machine learning models. Consequently, we performed one-hot encoding at two times to variables in status of renal disease, sex. First, the sex code underwent one-hot encoding, with 1 representing male sex and 2 representing female sex. Next, status of renal disease underwent one-hot encoding as 1 if a specific diagnostic code was assigned within 2 days of hospitalization and 0 otherwise. Finally, the entire of categorical variables were transformed as numerical vectors can be used in machine learning model.

### Imputation

Data cleaning was performed for each model, since the appropriate method differs depending on the type of model. The XGBoost and Random Forest models are a decision-tree-based ensemble machine learning algorithm, but artificial neural network and linear regression models are not. Therefore, we performed two imputation methods to preprocess missing values in numeric variables, by considering whether the model was a decision-tree-based model. Because the tree-based models can automatically handle missing values and are not sensitive to missing values or outliers^27^, any missing data was replaced with minus 1 in XGBoost and Random Forest models. Mean imputation would be appropriate to deal with the missing data for artificial neural network and linear regression model, because the numeric variables used in this study do not have a wide range of values. Accordingly, we performed mean imputation that convert missing values into mean values of the specific variable in artificial neural network and linear regression model.

### Normalization

Finally, we conducted normalization in artificial neural network and linear regression models and all variables were normalized using the minimum-maximum scaling method^28^, resulting in a range from 0 to 1.

### Data split

The AMC dataset (n = 3168) was separated as 80% for the training set (n = 2534) and 20% for the internal validation set (n = 634) using random split method. Additionally, we performed external validation to evaluate the generalizability of the model to the external validation set (n = 891) from the MIMIC-III.

### Model development and validation

The following models were developed: artificial neural network (ANN)^29^, linear regression^30^, extreme gradient boosting (XGBoost)^31^, and random forest^32^ models. These models were trained in training set including 17 clinical features and predicted a warfarin discharge dosage. We conducted Grid Search^33^ with random shuffles of 5-fold cross-validation^34^ to identify the optimal hyperparameters for each model. The entire of final hyperparameters of four models was shown in Supplementary Table S3. Finally, the XGBoost and random forest models utilized the raw dataset as the input, Whereas the ANN and linear regression models used the minimum-maximum scaled dataset as the input to help a rapid optimization of each models.

### Performance metrics

We used both of MAE and predictive accuracy as performance metrics to evaluate the model prediction ability. First, the discharge dosage of warfarin set as target. Then, the error between the predictions, including physicians initial dose and model prediction, and the discharge warfarin dosage were calculated for each sample. Finally, the errors for each sample were summed and divided with entire sample size to obtain the MAE. The MAE of the models was compared with the physicians initial dose to examine that our models’ predictions were more accurate and rapid than the physicians.

Subsequently, three thresholds set according to the suggestion of cardiovascular physicians that if the prediction error was within 0.5 mg, 1.0 mg, 1.5 mg, the prediction could be accepted as good, normal, the least, respectively. Reflecting this, we calculated the accuracy of the model prediction using three thresholds: 0.5 mg, 1.0 mg, and 1.5 mg. This approach calculating the accuracy was consulted a logistic regression that conducts a binary classification whether the prediction was greater than a specific cut-off value^35^. The prediction was classified as accurate if the MAE of the sample was smaller than the corresponding threshold; otherwise, it was classified as inaccurate. For example, when the threshold was 0.5 mg, if the MAE of a particular sample was 0.3 mg, the prediction was classified as accurate. We calculated the proportion of samples with accurate predictions determined by each model and evaluated the accuracy of predictions based on each threshold.

### Model interpretations

We used the Shapley additive explanations (SHAP) method to obtain insights of the predictions of our models and understand how each variable contributes to predictions^36^. The SHAP method is an explainable artificial intelligence method that decomposes the output of the model into the contributions of each feature, allowing for an analysis of the influence of each feature on the model^37^. It considers dependencies between features and can calculate positive and negative impacts, unlike traditional variable importance measures. Higher SHAP values indicate that the patient needs higher warfarin dose. The SHAP values calculated using the internal validation set were applied to visualize beeswarm and waterfall plots. First, the impact of each feature for model predictions was analyzed through a beeswarm plot. Second, waterfall plots showed that how the model considers the worth of each feature for individual predictions.

### Comparison of models’ and physicians’ predictions

We selected 20 data points with accurate model predictions, high physician prediction errors and 20 data points with high model prediction errors and accurate physician predictions. The XGBoost model was used. Subsequently, we constructed a dataset with 50% models accuracy. Next, we distributed these datasets to five physicians and asked them to predict the appropriate warfarin discharge dosage. We analyzed intraclass correlation coefficient (ICC) of the physicians’ predictions to test the interrater agreement using 2-way random effects model in R. Finally, we compared the predictions of the machine learning models and those of the physicians.

## Results

### Participants characteristics

The baseline characteristics of the two datasets used for model development and validation are listed in Table 1. Also, we additionally confirmed both of the last INR(PT) value and the duration of hospitalization for check the baseline condition of patients, but not used in the models.

**Table 1:** Characteristics of participants.

	AMC dataset (n = 3168)	MIMIC-III dataset (n = 891)
Demographics		
Age, mean (SD), years	62.3 (12.5)	65.3 (14.3)
Male	1674 (52.8%)	510 (57.2%)
Female	1494 (47.2%)	381 (42.8%)
Height, mean (SD), cm	162.2 (9.5)	171.0 (10.7)
Weight, mean (SD), kg	63.5 (13.0)	87.6 (46.1)
Lab events		
First INR(PT), mean (SD), s	1.7 (0.7)	1.7 (0.8)
Hemoglobin, mean (SD), g/dL	10.8 (1.9)	10.8 (1.9)
Platelet, mean (SD), K/uL	153.0 (71.2)	215.7 (106.0)
Vital Signs, (mean (SD), mmHg)		
Systolic blood pressure	115.9 (20.3)	115.0 (22.3)
Diastolic blood pressure	67.4 (12.2)	58.6 (1.4)
Status of disease (n, %)		
Renal disease	303 (9.6%)	292 (32.8%)
Other medication use, mean (SD), mg		
Aldosterone antagonist	54.3 (36.8)	34.4 (18.8)
Amiodarone	776.6 (810.0)	373.7 (245.7)
Aspirin	194.9 (67.7)	290.7 (192.2)
Diuretic, Loop	67.5 (109.0)	80.7 (119.1)
Lipid-lowering agents	27.7 (50.3)	77.9 (113.7)
Nitrate	97.1 (82.8)	4.2 (8.8)
Tramadol	148.7 (90.7)	95.0 (37.1)
Baseline		
Last INR(PT), mean (SD), s	2.2 (0.5)	2.2 (0.8)
Duration of hospitalization, mean (SD), days	14.4 (20.9)	8.2 (8.5)

The categorical variables, such as sex, status of disease, are presented as numbers and percentages of patients with a specific sex, diagnosis, and medication, respectively. The remaining numeric variables are presented as the mean and standard deviation. The lipid-lowering mediciation included statin family of drugs.

*SD* standard deviation, *TIA* transient ischemic attack, *INR* international normalized ratio, *PT* prothrombin time.

### Model performance

**Figure 3:**
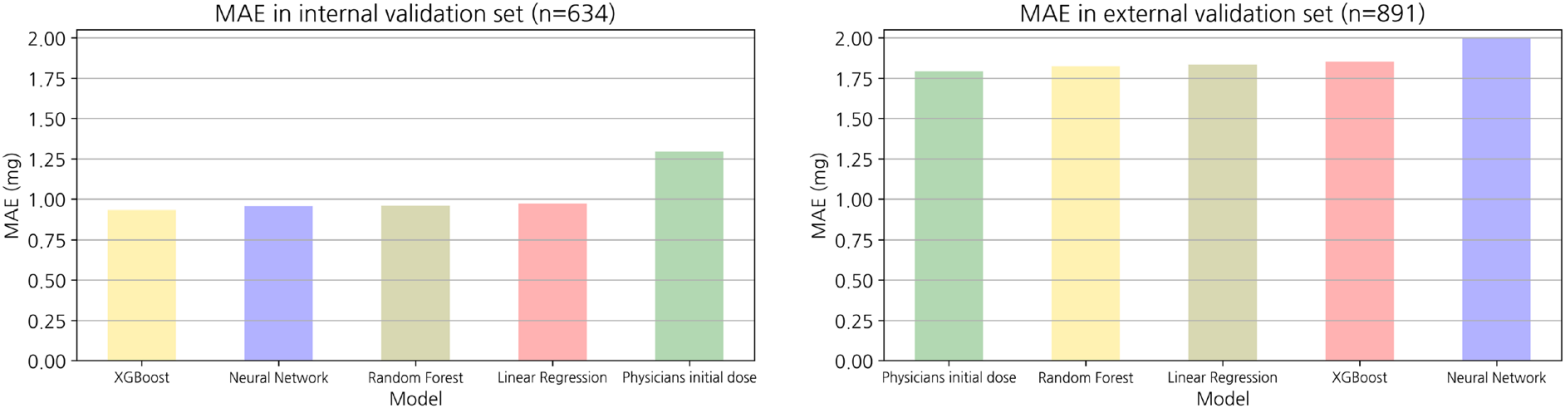
Bar plot representing the MAE. Performance abilities of the models based on the MAE were visualized using the internal validation (n = 634) and external validation (n = 891) sets. The unit of error is milligram (mg).

The MAE and accuracy at the threshold for both datasets are listed in Table 2. The following MAEs were calculated for the internal validation set: XGBoost, 0.9; random forest, 1.0; Artifical neural nets (ANN), 0.9; and linear regression, 1.0. All models had better prediction performance than expert physicians (MAE of 1.3). Using the external validation dataset, the following MAEs were achieved: physicians, 1.8; random forest, 1.8; linear regression, 1.8; ANN, 2.0; and XGBoost, 1.9. Consequently, internal validation of the internal validation set from the AMC EMRs confirmed that all predictions of the artificial intelligence models had lower errors and higher accuracy than those made by physicians regarding MAEs and accuracy. However, the physicians showed similar or superior performance when compared with all machine learning models in terms of the MAE, in external validation derived from the MIMIC-III. The MAE box plots of the internal validation set and external validation set are shown in Fig. 3.

**Table 2:** Model performance according to the MAE and accuracy.

	Internal validation set (n = 634)				External validation set (n = 891)			
	MAE	Accuracy (e = 0.5 mg)	Accuracy (e = 1.0 mg)	Accuracy (e = 1.5 mg)	MAE	Accuracy (e = 0.5 mg)	Accuracy (e = 1.0 mg)	Accuracy (e = 1.5 mg)
XGBoost	0.9	50.1	72.4	82.8	1.8	35.6	45.5	56.8
Random forest	1.0	49.1	69.6	81.2	1.8	35.1	44.6	56.6
Artificial neural net	0.9	49.1	70.8	84.4	2.0	30.2	47.8	58.8
Linear regression	1.0	46.5	69.2	85.0	1.8	26.4	48.9	48.9
Physicians initial dose	1.3	32.2	57.3	69.4	1.8	37.1	48.3	53.5

We conducted model performance evaluations of the internal validation set and external validation using the MAE and calculated the model prediction accuracy using three thresholds (0.5 mg, 1.0 mg, 1.5 mg). MAE, mean absolute error.

### Model interpretations

**Figure 4:**
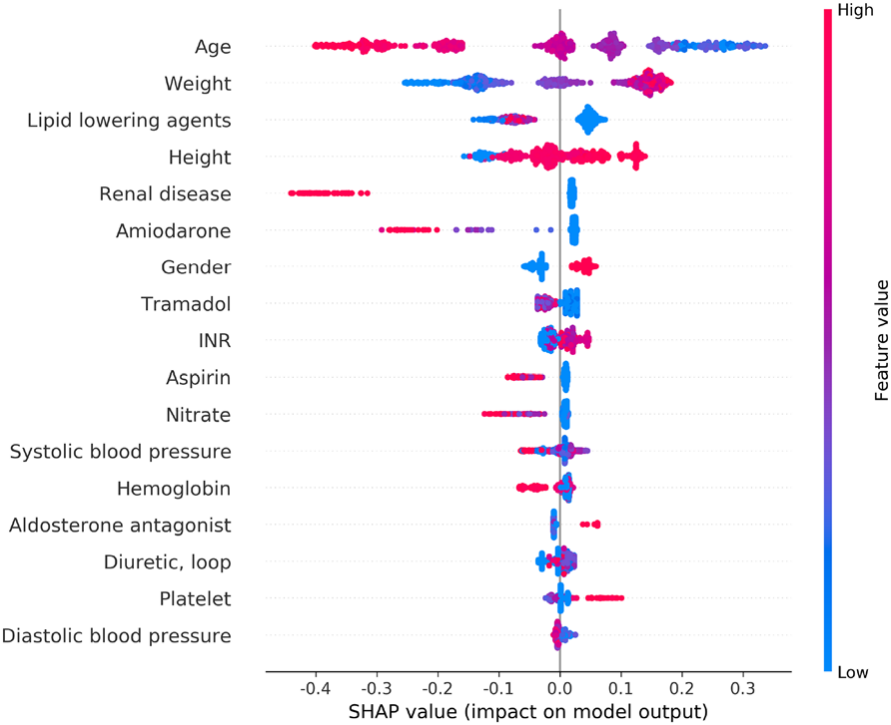
SHAP beeswarm plot of the features affecting the predictions of the XGBoost model. Features are ranked in descending order based on the absolute value of their influence on the XGBoost model. The x-axis indicates SHAP values. Each dot denotes a data point. Colors represent high values (red) or low values (blue) of specific data points. *SHAP* Shapley additive explanations.

**Figure 5:**
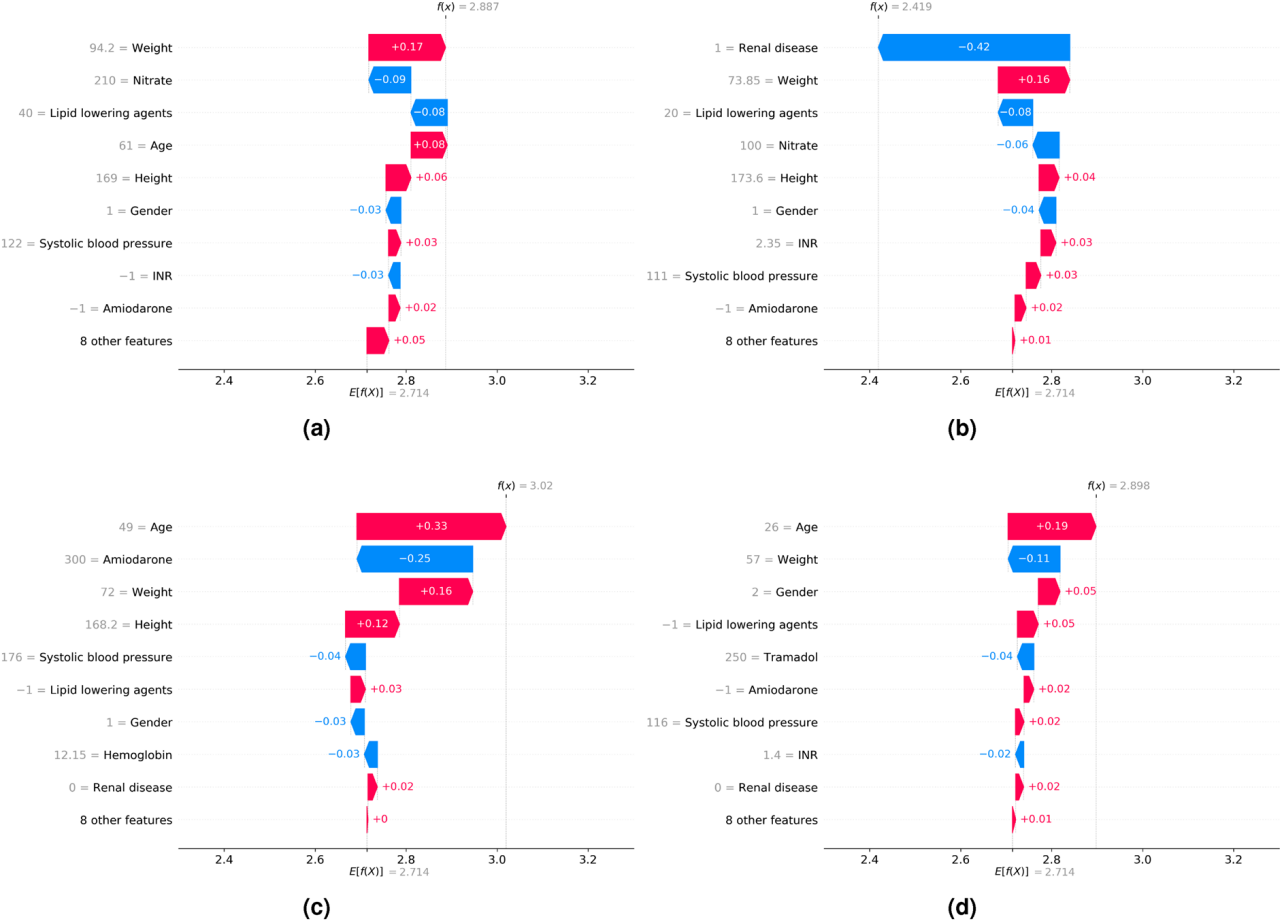
SHAP waterfall plot. The x-axis represents the individual warfarin dosage prediction of the models. The y-axis represents the input features of the models. E[f(x)] (2.714) represents the baseline value, which is the model output of the entire dataset, and f(x) represents the individual model output for each patient. Each arrow indicates whether a specific feature increased (red) or decreased (blue) the warfarin dosage. *SHAP* Shapley additive explanations.

We examined the SHAP values of the features with the most impact on model predictions using the beeswarm plot (Fig. 4). We also investigated the impact of features on individual predictions. We randomly selected 4 data points from the internal validation set with no missing values, and our models accurately predicted all of them. Subsequently, we calculated the SHAP values using a waterfall plot to explain individual predictions. The waterfall plot explains the influence of each feature on individual predictions (Fig. 5). The patient of Fig. 5a was 61-years-old male and taken with variable medications including aldosterone antagonist, nitrate, lipid lowering agents, diuretic. His height was 169cm and weight was 94.2 kg. The patients of Fig. 5b was 64-years-old male and taken nitrate and lipid lowering agents, also have a renal disease. His height was 173.6 cm and weight was 73.85 kg. The patient of Fig. 5c was 49-years-old male and taken with Amiodarone. His height was 168.2 cm and weight was 72 kg. The patient of Fig. 5d was 26-years-old female and taken with aldosterone antagonist, diuretic, nitrate, tramadol. Her height was 165.7 cm and weight was 57 kg. Especially, her systolic and diastolic blood pressure were 116 mmHg and 80 mmHg, respectively. As a result, we identified that the contributions of features affected a model prediction were different for each patient. For example, weight affected negative impact for patient of Fig. 5d, but positive impact to the rest of patients.

### Comparison of models’ and physicians’ predictions

**Figure 6:**
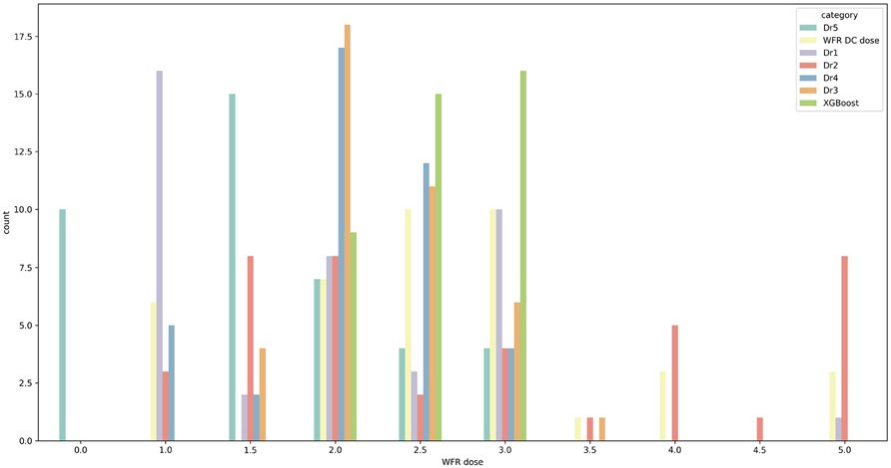
Distribution of warfarin dosage predicted by the physicians and models. We identified the predictions of five physicians and that of our model using 40 data points. The actual warfarin dosage at discharge (yellow) was distributed evenly within 1–5 mg. The average prediction dosage of our model (green) was 2 $.$ 6 mg, and the maximum prediction dosage of our model was 3 mg. The prediction accuracy of model and the physicians are 50% and 23%, respectively. *WFR* warfarin, *DC* discharge.

Finally, we collected the predictions of the model and those of the five physicians for 40 data points (Fig. 6). First, intraclass correlation coefficient (ICC) of the physicians’ predictions was calculated to measure the interrater agreement (Table 3). ICC is a test that how different raters measure subjects similarly from equal data point. It is important as it represents measurement errors caused by variation in rater judgement^38^. ICC values range from 0 to 1, with values less than 0.5 indicating low reliability, values between 0.5 and 0.75 indicating moderate reliability, values between 0.75 and 0.9 indicating good reliability, and values above 0.9 indicating excellent reliability^17^. In other words, the high reliability demonstrates less prone to prediction error based on raters. As shown in Table 3, the ICC value of five physicians was obtained to 0.36. It showed the significant variability in the distribution of dosage predicted by physicians, since the ICC value below 0.5. As shown in Fig. 6, it suggested that each physician tends to focus on a specific warfarin dosage range based on their clinical experience. Besides, prediction accuracy of the models and the five physicians was compared. Physicians accurately predicted approximately 9 of 40 samples, achieving 23% accuracy; however, the models demonstrated 50% accuracy. This indicated that it can be difficult for physicians to determine the appropriate warfarin discharge dosage based on the 17 clinical features obtained within 2 days of hospitalization.

**Table 3:** ICC of the predictions by the physicians for 40 data points.

	Intraclass correlation coefficient (95% CI)	p-value
Five physicians’ prediction	0.36 (0.19–0.54)	2.2e−14

## Discussion

In order to maximize the efficacy and safety of warfarin, it is important to determine the appropriate warfarin dosage for each individual^39^. However, there are variability in warfarin response for individual, due to differences about genetic and clinical factors. In particular, VKORC1, CYP2C9, and CYP4F2 polymorphisms account for approximately 40% of the variability in warfarin response, and novel genetic variants that have not yet been identified are assumed to account for approximately 50% of the variability. In contrast, clinical factors that can be considered in clinical practice only affect warfarin response by about 10%^40^. Nonetheless, genetic test is not routinely performed to determine the warfarin because genetic testing is costly and time-consuming^41^. On the other hand, warfarin has very reasonable cost in Korea. Therefore, the trial and error method that tries the specific warfarin dose then adjusted the dose has generally been used and is more efficient than genetic test. In clinical practice, physicians do not consider the genetic characteristics of patients rigorously^42^ and mainly decided the initial warfarin dosage through the frequent INR monitoring to maintain the target INR (2.0-3.0). Accordingly, we designed the machine learning models that used only clinical factors and predicted warfarin dose at discharge, to reflect the medical environment in Korea. Also, our models proposed in this study can be adoptable for developing countries where have difficult to conduct the genetic test. Additionally, the complex correlation with warfarin, including age, gender, weight, and drug-drug interactions, should be considered for warfarin. However, the priorities of features that have impact on warfarin are different as the health conditions of each patient are different, such as concurrent medication or disease status. Eventually, physicians depend on own clinical experiences to decide the initial warfarin dosage. We required five physicians who prescribed the warfarin at least 3 years predict the initial warfarin dosage for 40 data points to identify the difference of judgement of warfarin dosage for each physician. As a result, the ICC value analyzed in this study was 0.36, showing significant variability in the warfarin dose prediction ﻿distribution for each physician. Besides, the predictive accuracy of the physicians was less than half our XGBoost model. Consequently, our models can support the physicians by providing more objective and accurate warfarin dosage.

Our models showed similar or superior performance when compared to other warfarin dosage models (Table 4). We identified previous regression models that predict warfarin dosage as numerical target and use MAE as a performance metrics, to compare accurately between our models and other models. Gage et al developed a multiple regression model that predict warfarin dosage in derivation cohort (N = 1015), included Caucasian and African American, Hispanic, and validated the models in validation cohort (N = 292)^5^. Both of pharmacogenetic and clinical model were developed and reached a MAE of 1.0, 1.5, respectively. Pavani et al developed an artificial neural network using ten genetic variables as inputs and therapeutic warfarin dosage as the output in Indian population (N = 240)^6^. Roche-Lima et al collected cardiovascular patients of 190 Caribbean Hispanic were > 21 years old and developed seven machine learning algorithms that predict warfarin dosing in Caribbean Hispanics using pharmacogenetic data^7^. Among them, random forest regressor (RFR) significantly outperformed all other models with a MAE of 4.74. Tong et al recruited 685 patients who diseased atrial fibrillation or thromboembolic venous disease in a Spanish population using the data last 3 consecutive months and used multiple linear regression^8^. Both of pharmacogenetic and clinical model were developed and internally validated with a MAE of 3.5, 5.0, respectively in a validation cohort (N = 129). Grossi et al collected 377 patients who were over 18 years old and treated with warfarin in Caucasian population and developed an artificial neural network to predict an optimal warfarin maintenance dose^9^. The final model reached a MAE of 5.72. Saleh recruited 4271 multi-ethnicity patients who received warfarin and developed an artificial neural network using both of genotyping and clinical data. The artificial neural network model reached a MAE of 9.0. Hernandez et al generated pharmacogenomic warfarin dosing model using clinical and genotyping retrospective data from a derivation cohort of 349 African Americans patients were $\ge$ 18 years and the model reached with a MAE of 10.9 mg/week^11^. Alzubiedi et al collected demographic, clinical, and genetic data from 163 African-American patients with a stable warfarin dose^12^. They developed both of a multiple linear regression model and artificial neural network model with MAE of 10.8, 10.9 respectively. Whereas, both of XGBoost and artificial neural network model in this study achieved 0.9 with MAE and outperformed aforementioned algorithms. Additionally, our models provided more appropriate warfarin dosage than those initially prescribed by physicians using clinical data within 2 days of hospitalization. It demonstrated that the models can make appropriate warfarin dosing decisions without the same level of effort as physicians who would consider various factors such as the INR value. These results are likely the results of successfully selecting important variables that may interact with warfarin from our initial clinical data obtained through discussions with experienced clinical experts and effectively utilizing the refined variables.

**Table 4:** Comparison with prior works.

	Number of data instances	Features	Study population	Algorithm	MAE
Choi et al*	3168	Clinical	South Korean	XGBoost	0.9
Choi et al*	3168	Clinical	South Korean	ANN	0.9
Choi et al	3168	Clinical	South Korean	Random Forest	1.0
Choi et al	3168	Clinical	South Korean	Linear Regression	1.0
Gage et al^5^	1307	Clinical + pharmacogenetic	Caucasian, African American	Regression	1.0
Gage et al.^5^	1307	Clinical	Caucasian, African American	Regression	1.5
Pavani et al.^6^	240	Clinical + pharmacogenetic	Indian	ANN	1.97
Roche-Lima et al.^7^	190	Clinical + pharmacogenetic	Caribbean Hispanics	Random forest	4.7
Tong et al.^8^	685	Clinical + pharmacogenetic	Spanish	Multiple linear regression	3.5
Tong et al.^8^	685	Clinical	Spanish	Multiple linear regression	5.0
Grossi et al.^9^	377	Clinical + pharmacogenetic	Caucasian	ANN	5.72
Saleh et al.^10^	4271	Clinical + pharmacogenetic	Multi-ethnicity	ANN	9.0
Hernandez et al.^11^	349	Clinical + pharmacogenetic	African–American	Multivariate regression	10.9
Alzubiedi et al.^12^	163	Clinical + pharmacogenetic	African–American	Linear regression	10.8
Alzubiedi et al.^12^	163	Clinical + pharmacogenetic	African–American	ANN	10.9

Our models were compared with previous models that developed using both of genetic and clinical data or only clinical data. Performance metric was used MAE. The information of study population’ ethnicity was included. The number of data participants included the number of model development and validation cohort.

*Represents the best performance model.

*ANN* artificial neural network.

## Limitation

Our study has a limitation of homogeneous caused its single-center design. We suffered the ethical issues of obtaining the multi-center EMRs. It is difficult to access to EMRs to different centers, because it includes patient’s medical record and cover patient privacy. Eventually, we had no choice about the diverse populations, but to should use a single population. It caused that the external validation in this study didn’t successfully perform and our models can’t be generalized to the different ethnicity. In the further work, we have to obtain an access of a multi-center cohort and conduct a multi-center study, to improve the accuracy and representativeness of the models. Additionally, if we obtain the data with larger sample size, it would be proper separating the group by low-dose and high-dose, respectively and training the models to improve the accuracy of the models.

## Conclusion

The patients who participated in this study hospitalized for an average 14 days and the INR measurement of the first and last days of hospitalization were 1.7 and 2.2, respectively. In other words, we assumed that the discharge warfarin dosage was appropriate as the initial dosage since the discharge dosage make patients maintain the target INR (from 2.0 to 3.0). Finally, we developed 3 machine learning models and 1 deep learning model using data of a model development cohort from a tertiary hospital in Korea to predict warfarin discharge dosage. Then, we evaluated our models with MAE and accuracy in both of internal and external validation. Our models not only outperformed physicians in internal validation, but also the previous models. In internal validation set with MAE, XGBoost and artificial neural network models achieved 0.9, and random forest and linear regression models achieved 1.0, whereas physicians achieved 1.3. Besides, our models outperformed previous warfarin dosing algorithm as we mentioned. Therefore, our models that provide proper warfarin dose within 2 days of hospitalization can be useful for patients when just start warfarin and might be effective tools that help physicians choose the personalized warfarin dosage when initiation warfarin.

### Supplementary Information

Supplementary Tables.

## Data Availability

The data that support the findings of this study are available from the corresponding author on reasonable request, due to ethical concerns and confidentiality agreements.
