# Supplementary material for: Machine learning models to predict the warfarin discharge dosage using clinical information of inpatients from South Korea
Source: Sci Rep. 2023 Dec 18;13:22461. doi: 10.1038/s41598-023-49831-6 (PMC10725866; doi:10.1038/s41598-023-49831-6)
Supplement: Supplementary file 1 — Supplementary Tables. [file 41598_2023_49831_MOESM1_ESM.pdf]

Machine learning models to predict the warfarin discharge dosage using clinical information of East Asian inpatients

Heejung Choi[1], Hee Jun Kang[2], Imjin Ahn[3], Hansle Gwon[3], Yunha Kim[3], Hyeram Seo[1], Ha Na Cho[3], JiYe Han[1], Minkyoung Kim[1], Gaeun Kee[3], Seohyun Park[3], Osung Kwon[4], Jae-Hyung Roh[5], Ah-Ram Kim[6], Ju Hyeon Kim[7], Tae Joon Jun[8, +], Young-Hak Kim[9, +,*]

[1]Department of Medical Science, Asan Medical Institute of Convergence Science and Technology, Asan Medical Center, University of Ulsan College of Medicine, 88, Olympic-ro 43 gil, Songpa-gu, 05505, Seoul, Republic of Korea

[2]Division of Cardiology, Asan Medical Center, 88, Olympicro 43gil, Songpagu, 05505, Seoul, Republic of Korea

[3]Department of Information Medicine, Asan Medical Center, 88, Olympicro 43gil, Songpagu, 05505, Seoul, Republic of Korea

[4]Division of Cardiology Department of Internal Medicine, Eunpyeong St Mary's Hospital, Catholic University of Korea, Seoul, Republic of Korea

[5]Department of Internal Medicine, Chungnam National University College of Medicine, Chungnam National University Sejong Hospital, 20, Bodeum 7-ro, Sejong-si , 30099), Sejong, Republic of Korea

[6]Division of Cardiology, Department of Internal Medicine, Asan Medical Center, University of Ulsan College of Medicine, Seoul, Republic of Korea

[7]Department of Cardiology, Cardiovascular Center, Korea University Anam Hospital, Korea University College of Medicine, 73, Goryeodae-ro, Seongbuk-gu, Seoul 02841, South Korea

8Big Data Research Center, Asan Institute for Life Sciences, Asan Medical Center, 88, Olympicro 43gil, Songpagu, 05505, Seoul, Republic of Korea

[9]Division of Cardiology, Department of Information Medicine, Asan Medical Center, University of Ulsan College of Medicine, 88, Olympicro 43gil, Songpagu, 05505, Seoul, Republic of Korea

[+]these authors contributed equally to this work

[*]email: mdyhkim@amc.seoul.kr

Corresponding author:

Young-Hak Kim, MD, PhD

Division of Cardiology, University of Ulsan College of Medicine

88, Olympic-ro 43 gil, Songpa-gu, Seoul 05505, Korea

Phone: 82 2 301 0 3955

Supplementary Table S1. ICD-10 diagnostic codes used to categorize diseases. We used only three or four strings from the front.

Diagnosis	ICD-10 code
Renal disease	N03, N04, N05, N10, N11, N12, N13, N14, N15, N16, N17, N18, N19, Z49, Z940, Z992

Supplementary Table S2. Information of the four models hyperparameter.

Model	Hyper-parameter
**XGBoost**	Number of estimators: 100<br>Learning rate: 0.1<br>Max depth: 2<br>Objective function: regression with squared log loss<br>Minimum sum of instance weight needed in a child: 10<br>L1 regularization term on weights: 0.1<br>Minimum loss reduction required to make a further partition on a leaf node of the tree: 0
**Random Forest**	Number of estimators: 400<br>Max depth: 5<br>Max leaf nodes: 9<br>Minimum of leaf: 4<br>Number of samples to draw from dataset to train each base estimator: 0.7<br>Minimum of impurity decrease to split the nodes: 0.0005<br>Number of minimum samples to split nodes: 5<br>Minimum weighted fraction of the sum total of weights (of all the input samples) required to be at a leaf node: 0.4
**Artificial neural network**	Activation function: identity function<br>Hidden layer: (600, 400, 300, 300)<br>Learning rate: 0.01<br>Solver: Minibatch gradient descent<br>Batch size: 16<br>Max iteration: 300
**Linear Regression**	Intercept: used
